# Influence of wall distensibility on local hemodynamics at the normal carotid bifurcation: a fluid–structure interaction study

**DOI:** 10.1007/s10237-026-02103-4

**Published:** 2026-07-21

**Authors:** Sara Zambon, Valentina Mazzi, Karol Calò, Mariachiara Arminio, Sabrina Nocerino, Claudio Chiastra, David A. Steinman, Umberto Morbiducci, Diego Gallo

**Affiliations:** 1 PolitoBIOMed Lab, Department of Mechanical and Aerospace Engineering, Politecnico di Torino, Corso Duca degli Abruzzi 24, 10129 Turin, Italy; 2 Biomedical Simulation Laboratory, Department of Mechanical & Industrial Engineering, University of Toronto, Toronto, ON Canada

**Keywords:** Carotid artery, Computational fluid dynamics, Prestress, Collagen fibers, Wall shear stress, Helicity

## Abstract

The role of local hemodynamics on atherosclerosis at the carotid bifurcation has been the subject of study by computational fluid-dynamics (CFD) simulations for over three decades. Nevertheless, questions still swirl about the inherent rigid-wall assumption, especially with the introduction of increasingly predictive—but also increasingly intricate—hemodynamic parameters. Two-way-coupled fluid–structure interaction (FSI) simulations were performed on a cohort of 10 carotid bifurcations with ostensibly normal lumen geometries, along with CFD simulations assuming rigid arterial walls. In FSI simulations, carotid wall mechanical properties were assumed to be anisotropic via a fiber-reinforced hyperelastic material model, also accounting for prestress and external tissue support. Three-element Windkessel models were used to impose pressure conditions consistent with patient-specific measured inflow rates and outflow divisions. Maximum cross-sectional area changes over the cardiac cycle were generally less than 21%. Qualitatively, only small-to-moderate differences were observed between FSI and CFD simulations in terms of wall shear stress (WSS) and intravascular flow patterns. Quantitatively, median differences in the surface areas exposed to low time-averaged WSS (TAWSS), high oscillatory shear index (OSI) and topological shear variation index (TSVI) were 4.1%, 1.4%, and 2.3%, respectively, and co-localized satisfactorily (median similarity index: 0.83, 0.79, and 0.68, respectively). CFD simulations assuming arterial rigid walls are generally sufficient to adequately capture hemodynamic features of biological/clinical relevance, even for sensitive quantities like TSVI. However, the benefits of FSI for computation of structural quantities and the ability to explore the synergistic relationship between these quantities and hemodynamic stresses on the endothelium should not be overlooked.

**Supplementary Information:**

The online version contains supplementary material available at https://doi.org/10.1007/s10237-026-02103-4.

## Introduction

Atherosclerosis is a lipid-driven chronic inflammatory disease representing a leading cause of death globally. It is widely recognized that the biomechanical environment plays a crucial role both at the early-stage of the atherosclerotic disease and in its development (Malek et al. 1999; Humphrey 2009; Friedman et al. 2010), with a tendency for plaques to develop where blood flow patterns are most complex, as in arterial bifurcations (Morbiducci et al. 2016), bends, and branches (Friedman et al. 1983). In this context, the carotid bifurcation is a common site for fluid mechanics studies on the complex interplay between blood flow and vascular pathophysiology, due to its intricate hemodynamics and its preferential atherosclerosis development. Most studies have focused on the role of wall shear stress (WSS) due to its association with endothelium function and with flow disturbances such as flow stagnation, separation and recirculation (Morbiducci et al. 2016). Recently, WSS topological skeleton has emerged as an independent predictor of markers of vascular disease, including intima-media thickness in carotid bifurcations at 5-year follow-up after endarterectomy (Morbiducci et al. 2020), and wall thickness changes over time on swine models (Mazzi et al. 2021). Furthermore, distinct WSS topological features on the luminal surface of the carotid bifurcation are instrumental in providing a template for blood-wall mass transfer of biochemicals (Farghadan and Arzani 2019), in particular low-density lipoproteins (Mazzi et al. 2022), which play a relevant role in the initiation of atherosclerosis.

In addition to near-wall flows, intravascular flow features are recognized to play a relevant role in the onset of carotid bifurcation atherosclerosis. Previous studies on the carotid bifurcation hemodynamics have provided evidence on (i) the link between the volume of recirculating blood, a feature of disturbed flow, and atherosclerotic biomarkers (Martorell et al. 2014), and (ii) the atheroprotective role of helical blood flow in suppressing flow disturbances (Gallo et al. 2012).

Since the late 1990s, image-based computational fluid dynamics (CFD) has been established as an effective tool for studying hemodynamics with high spatiotemporal resolution in personalized in silico models. However, traditional CFD studies almost invariably adopt a rigid-wall assumption, which may overlook the influence of wall motion on local flow patterns, therefore introducing idealizations that potentially limit the accuracy of the results. In contrast, fluid–structure interaction (FSI) approaches simulate wall distensibility by coupling blood flow dynamics with arterial wall mechanics (Hou et al. 2012; Schwarz et al. 2023), providing a more realistic depiction of the hemodynamics in the carotid bifurcation (Malvè et al. 2014; Lopes et al. 2020; Bantwal et al. 2021; Aryan et al. 2025). However, FSI models are characterized by increased complexity and computational cost with respect to rigid-wall CFD. Moreover, their accuracy is further challenged by the inherent heterogeneity of the arterial wall (Hariton et al. 2007b; Alastrué et al. 2010), which exhibits a highly nonlinear and anisotropic mechanical behavior.

This study investigates the effect of wall distensibility on carotid bifurcation hemodynamics, assessed through both established and emerging WSS-based quantities, as well as intravascular flow features such as helical flow and flow recirculation. To this end, ten subject-specific models of ostensibly healthy human carotid bifurcations were analyzed with paired rigid-wall CFD and with fully coupled FSI simulations. The latter approach incorporated (i) a fiber-reinforced model of the arterial wall, capturing its anisotropic behavior by embedding collagen fibers in an isotropic continuum (Holzapfel et al. 2000), (ii) the estimation of the vessel’s physiological loading state as introduced by Hsu and Bazilevs (2011), and (iii) viscoelastic support from the tissue surrounding the arterial wall (Moireau et al. 2012).

## Materials and methods

### Study participants and imaging

**Fig. 1:**
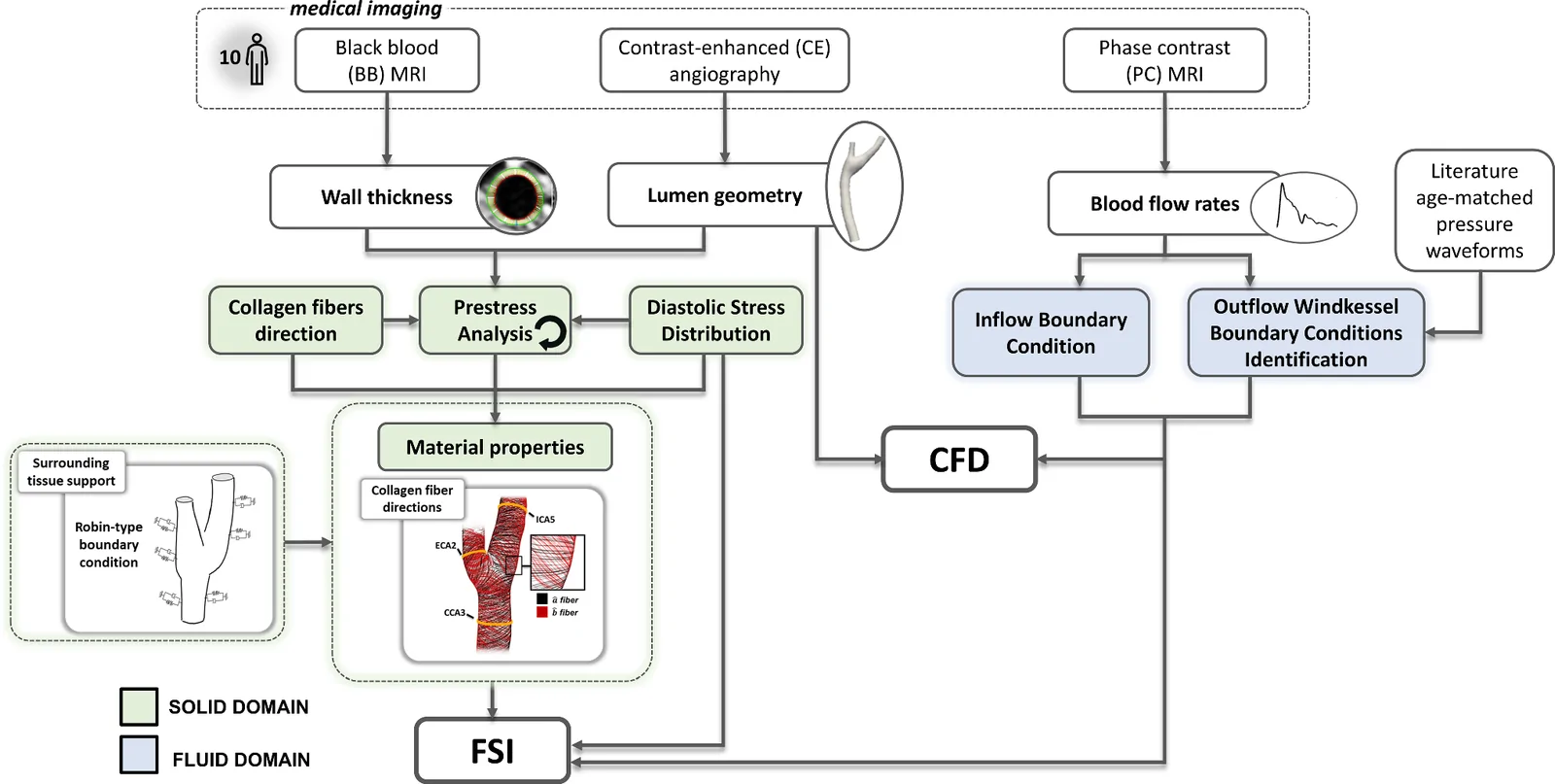
Schematic diagram of the study design illustrating the methodologies used to perform FSI and CFD simulations in patient-specific carotid bifurcations. Medical imaging data (black-blood MRI, contrast-enhanced angiography, and phase contrast MRI, Gallo et al. (2018)) are used to extract wall thickness, lumen geometry, and blood flow rates, respectively. These inputs inform the material properties, prestress analysis, and boundary conditions for the solid and fluid domains. The model incorporates a stress-driven methodology to determine collagen fiber orientation. Outflow boundary conditions are derived from measured flow rates and age-matched pressure waveforms from literature. CFD and FSI simulations are then performed to compare their results and assess the impact of wall distensibility on key hemodynamic features

An overview of the methods is provided in Fig. 1. The imaging data of ostensibly healthy right carotid bifurcation was acquired as part of a previous CFD-based analysis of hemodynamic risk factors for early atherosclerosis (Gallo et al. 2018). In that study individuals had undergone 3D contrast-enhanced magnetic resonance angiography (CE-MRA) and 2D phase contrast magnetic resonance imaging (PC-MRI), allowing measurement of individual 3D lumen geometry and time-varying inflow/outflow rates. The MRI exam also included pre- and post-contrast 2D edge-enhanced black-blood MRI slices acquired roughly 0.5 mm distal to the flow divider, transverse to the common carotid artery (CCA)–internal carotid artery (ICA) tract, i.e., nominally transverse to the carotid bulb, to obtain the inner and outer wall boundaries. From these the mean wall thickness of the ICA bulb was measured. Further details of the imaging, lumen geometry segmentation and wall thickness measurement protocols are detailed elsewhere (Wasserman et al. 2010; Hoi et al. 2010; Gallo et al. 2018). Institutional review board approval was previously obtained, and participants gave informed consent for use of their anonymized and deidentified data for research publications.

### Geometry reconstruction and discretization

For the present study, 10 cases were selected, ostensibly spanning the range of flow phenotypes from normal to disturbed. In detail, we identified one case from each decile of percent luminal surface area exposed to elevated relative residence time (RRT), as obtained from previous CFD simulations (Gallo et al. 2018). It is worth noting that in the original study by Gallo et al. (2018), care had already been taken to exclude cases with evidence of inward remodeling or wall irregularities, so while the cases were middle-aged to older, the lumen geometries could be considered representative of those from healthy young adults, for the present purposes.

Lumen geometries were clipped at least fifteen, seven and three radii from the bifurcation (Supplementary Fig. 1), respectively for the CCA, ICA, and external carotid artery (ECA) to minimize the effect of geometry truncation on the computed bifurcation flow patterns, as demonstrated in previous studies (Hoi et al. 2010; Gallo et al. 2015). The wall structural domain was obtained by outward extrusion of the lumen geometry by uniform but subject-specific wall thickness measured at the ICA bulb using Meshmixer (Autodesk, Inc.). The artery walls were assumed to have uniform thickness, but using case-specific wall thickness values previously averaged around each subject’s carotid bulb, as noted in Sect. 2.1. Age and measured wall thickness of the 10 selected cases are shown in Table 1.

**Table 1:** Age and mean wall thickness values measured at the internal carotid artery bulb by black-blood magnetic resonance imaging, used to prescribe uniform wall thickness for each case

Case number	Age (years)	Wall thickness (mm)
1	63	0.92
2	59	1.07
3	71	1.03
4	54	0.87
5	36	0.94
6	63	0.76
7	40	0.92
8	57	0.91
9	80	0.87
10	53	0.87

The fluid (lumen) and solid (wall) domains were discretized into unstructured tetrahedral grids using the TetGen library embedded in SimVascular (Si 2008; Updegrove et al. 2017) following a grid independence analysis. Specifically, for the fluid domain five spatial discretizations with a radius-based refinement strategy were considered, with maximum element size ranging from 0.275 mm to 0.125 mm, targeting both WSS-based and intravascular flow quantities. The selected grid yielded errors of less than 5% compared to the finest grid and had a maximum element size of 0.17 mm with two near-wall boundary layers, resulting in fluid grids ranging from 1.5 to 6.7 million elements (Supplementary Table 1). For the solid domain, four discretizations were tested, with maximum element size ranging from 0.7 mm to 0.1725 mm. The grid with a maximum edge size of 0.4 mm was selected, as the error in maximum Cauchy principal stress (MPS) compared to the finest grid was less than 5%, leading to solid meshes ranging from 289,000 to 770,000 elements (Supplementary Table 2). At the fluid–solid interface, nodal matching satisfied kinematic and dynamic interfacial constraints.

### Fluid–structure interaction simulations

Numerical simulations were conducted using the monolithic finite-element based solver of the SimVascular package, svFSI (Zhu et al. 2022; Lan et al. 2018), based on the Arbitrary Lagrangian–Eulerian formulation (ALE) to model the interaction between fluid and solid domains. Computational resources were provided by HPC@POLITO (http://www.hpc.polito.it) and CINECA under the ISCRA initiative.

#### Fluid domain–governing equations and boundary conditions

The governing equations of fluid motion under unsteady-state conditions for an assumed incompressible, homogeneous, Newtonian fluid, in the ALE formulation, take into account the deformability of the fluid domain ${\Omega}_\mathrm{f}(t)$ incorporating the grid velocity $\widehat{{\boldsymbol{v}}}$ into the convective term (Zhu et al. 2022; Schwarz et al. 2023):

1 $$\rho_{{\mathrm{f}}} \left[ {\frac{\partial {\boldsymbol{v}}}{{\partial t}} + \left( {\left( {{\boldsymbol{v}} - \hat{{\boldsymbol{v}}}} \right) \cdot \nabla } \right){\boldsymbol{v}}} \right] - \nabla \cdot {\boldsymbol{\sigma}}_{{\mathrm{f}}} = 0\,\, in\,\,  \Omega_{{\mathrm{f}}} \left( t \right)$$

2 $$\nabla \cdot {\boldsymbol{v}} = 0\,\, in \,\, \Omega_{{\mathrm{f}}} \left( t \right)$$

where ${\boldsymbol{v}}$ is the fluid velocity, $\rho_{{\mathrm{f}}}$ is the fluid density (equal to 1060 ${\mathrm{kg}}/{\mathrm{m}}^{3}$), and ${\boldsymbol{\sigma}}_{{\mathrm{f}}} = \mu_{{\mathrm{f}}} \left( {\nabla {\boldsymbol{v}} + \nabla {\boldsymbol{v}}^{T} } \right) - p\boldsymbol{I}$ is the Cauchy stress tensor, with $\mu_{{\mathrm{f}}}$ denoting the dynamic viscosity of the fluid (equal to 0.0035 $\mathrm{Pa} \cdot s$), p the pressure and $\boldsymbol{I}$ the identity tensor.

At the CCA inflow boundary, the MRI-measured patient-specific volumetric flow rates were prescribed in terms of parabolic velocity profiles (Moyle et al. 2006). A multidomain approach was applied at the ICA and ECA outflow boundaries, where a 3D-0D coupling scheme was adopted using three-element Windkessel models to account for the impedance of the downstream vasculature (Vignon-Clementel et al. 2006). The parameters of each Windkessel model were tuned to match subject-specific PC-MRI-measured cycle-averaged flow splits at the outlets and age-specific carotid pressure pulse (Hirata et al. 2006), using 1D modeling and MATLAB’s Optimization Toolbox (The MathWorks Inc., R2023b). Windkessel parameters are reported in Supplementary Table 3. For each case, the same Windkessel calibrated parameters were applied to both CFD and FSI simulations. We simulated 3 cardiac cycles to ensure cycle-to-cycle convergence of the results, verifying that the variation in systolic peak pressure between consecutive cycles was less than 1%.

#### Solid domain–governing equations and boundary conditions

The governing equation in the solid domain ${\Omega}_{s} \left( t \right)$ is given by:

3 $$\rho_{{\mathrm{s}}} \left( {\frac{{\partial \widehat{{{\boldsymbol{v}}}_{\mathrm{s}} }}}{\partial t} + \left( {\widehat{{{\boldsymbol{v}}_{{\mathrm{s}}} }} \cdot \nabla } \right) \widehat{{{\boldsymbol{v}}_{{\mathrm{s}}} }}} \right) = \nabla \cdot {\boldsymbol{\sigma}}_{{\mathrm{s}}} \,\, in\,\, \Omega_{{\mathrm{s}}} \left( t \right)$$

where $\rho_{{\mathrm{s}}}$ is the solid domain density (equal to 1200 ${\mathrm{kg}}/{\mathrm{m}}^{3}$), $\widehat{{{\boldsymbol{v}}_{{\mathrm{s}}} }} = \frac{{\partial {\boldsymbol{u}}_{{\mathrm{s}}} }}{\partial t}$ is the velocity vector of the solid domain with ${\boldsymbol{u}}_{{\mathrm{s}}}$ being the solid domain displacement vector field, and ${\boldsymbol{\sigma}}_{{\mathrm{s}}}$ is the Cauchy stress tensor. External body forces such as gravity or body motion were neglected.

At the fluid–solid interface ${\Gamma }\left( t \right)$, the solver enforces the following coupling conditions on tractions and velocities (Bäumler et al. 2020):

4 $${\boldsymbol{\sigma}}_{{\mathrm{f}}} \widehat{{{\boldsymbol{n}}_{{\mathrm{f}}} }} + {\boldsymbol{\sigma}}_{{\mathrm{s}}} \widehat{{{\boldsymbol{n}}_{{\mathrm{s}}} }} = 0$$

5 $$\widehat{{{\boldsymbol{v}}_{{\mathrm{s}}} }} = {\boldsymbol{v}}$$

with $\widehat{{{\boldsymbol{n}}_{{\mathrm{f}}} }}$ and $\widehat{{{\boldsymbol{n}}_{{\mathrm{s}}} }}$ being the normal directions at the fluid and solid domain interface, respectively (with $\widehat{{{\boldsymbol{n}}_{{\mathrm{s}}} }} = - \widehat{{{\boldsymbol{n}}_{{\mathrm{f}}} }}$). At CCA, ECA and ICA ring-shaped solid domain boundary sections, a homogeneous Dirichlet boundary condition (${\boldsymbol{u}}_{{\mathrm{s}}}$ = 0) was prescribed.

To account for the radial constraint on vessel dilatation imposed by the tissues surrounding the carotid artery (Liu et al. 2007), a Robin-type boundary condition was applied on the outer surface of the carotid wall ${\Gamma}_{{{\mathrm{ext}}}} \left( t \right)$. The applied Robin-type boundary condition, embedded in SimVascular (Bäumler et al. 2020), modeled the viscoelastic support provided by the surrounding tissues:

6 $${\boldsymbol{\sigma}}_{{\mathrm{s}}} \cdot \hat{{\boldsymbol{n}}} = - k {\boldsymbol{u}}_{{\mathrm{s}}} - c \frac{{\partial {\boldsymbol{u}}_{{\mathrm{s}}} }}{\partial t} - p_{0} \hat{{\boldsymbol{n}}}\,\, on\,\, \Gamma_{{{\mathrm{ext}}}} \left( t \right)$$

where ${\boldsymbol{\sigma}}_{{\mathrm{s}}} \cdot \hat{{\boldsymbol{n}}}$ is the traction vector derived projecting the Cauchy stress tensor ${\boldsymbol{\sigma}}_{{\mathrm{s}}}$ along the outward unit normal vector $\hat{\boldsymbol{n}}$ direction at ${\Gamma}_{{{\mathrm{ext}}}} \left( t \right)$, k is the stiffness coefficient, c is the viscous coefficient, and $p_{0}$ is the external pressure. The parameters in Eq. (6) were set according to previous studies (k = 10^7^ $\frac{N}{{m^{3} }}$, c = 2·10^3^ $\frac{N \cdot s}{{m^{3} }}$, $p_{0}$ = 0 Pa) (Pozzi et al. 2021).

#### Wall material model

The carotid wall was modeled as a homogeneous, nonlinear and anisotropic fiber-reinforced hyperelastic material adopting the Holzapfel–Gasser–Ogden (HGO) model (Holzapfel et al. 2000). Specifically, the mechanical behavior of the vessel wall was described through the strain energy function (SEF) Ψ:

7 $$\Psi = \Psi_{{{\mathrm{vol}}}} \left( J \right) + \overline{\Psi}_{{{\mathrm{iso}}}} \left( {\overline{{\boldsymbol{C}}}} \right) + \overline{\Psi}_{{{\mathrm{aniso}}}} \left( {\overline{{\boldsymbol{C}}}, \hat{{\boldsymbol{a}}}, \hat{{\boldsymbol{b}}}} \right)$$

where the volumetric component $\Psi_{{{\mathrm{vol}}}}$ serves as a dilatational penalty function to enforce the incompressibility constraint, and the two isochoric components $\overline{\Psi}_{{{\mathrm{iso}}}}$ and $\overline{\Psi}_{{{\mathrm{aniso}}}}$ represent the isotropic non-collagenous ground matrix and the nonlinear anisotropic contribution of the two embedded families of collagen fibers, respectively. In detail, the volumetric contribution in Eq. (7) is defined as follows:

8 $$\Psi_{{{\mathrm{vol}}}} \left( J \right) = \frac{1}{2}{\mathrm{K}}\left( {J - 1} \right)^{2}$$

where ${\mathrm{K}}$ is the bulk modulus and $J = \det {\boldsymbol{F}}$ is the determinant of the total deformation gradient tensor ${\boldsymbol{F}} = \left( {J^{\frac{1}{3}} {\boldsymbol{I}}} \right)\overline{\boldsymbol{F}}$, expressed in terms of the volumetric $\left( {J^{\frac{1}{3}} {\boldsymbol{I}}} \right)$ and isochoric ($\overline{{\boldsymbol{F}}}$) contributions (Gasser and Holzapfel 2002). As for the isochoric isotropic component $\overline{\Psi}_{{{\mathrm{iso}}}}$ in Eq. (7), it can be expressed as:

9 $$\overline{\Psi}_{{{\mathrm{iso}}}} \left( {\overline{{\boldsymbol{C}}}} \right) = \frac{c}{2} \left( {\overline{I}_{1} - 3} \right)$$

where c is the isotropic matrix shear modulus and $\overline{I}_{1} = {\mathrm{trace}}\left( {\overline{{\boldsymbol{C}}}} \right)$ is the first invariant of the symmetric modified right Cauchy-Green tensor $\overline{{\boldsymbol{C}}} = \overline{{\boldsymbol{F}}}^{T} \overline{{\boldsymbol{F}}}$. Finally, the isochoric anisotropic contribution $\overline{\Psi}_{{{\mathrm{aniso}}}}$ was formulated as follows:

10 $$\overline{\Psi}_{{{\mathrm{aniso}}}} \left( {\overline{\boldsymbol{C}}, \hat{\boldsymbol{a}}, \hat{\boldsymbol{b}}} \right) = \frac{{k_{1} }}{{2k_{2} }} \{ e^{{k_{2} \left[ {\overline{I}_{4} - 1} \right]^{2} }} - 1\} + \frac{{k_{1} }}{{2k_{2} }} \{ e^{{k_{2} \left[ {\overline{I}_{6} - 1} \right]^{2} }} - 1 \}$$

where the invariants $\overline{I}_{4} = \hat{\boldsymbol{a}} \cdot \overline{\boldsymbol{C}} \cdot \hat{\boldsymbol{a}}$ and $\overline{I}_{6} = \hat{\boldsymbol{b}} \cdot \overline{\boldsymbol{C}} \cdot \hat{\boldsymbol{b}}$ represent the square values of the stretch along the fibers direction indicated by the unit vectors $\hat{\user2{a}}$ and $\hat{\user2{b}}$, and $k_{1}$ and $k_{2}$ are strictly positive stress-like and nonlinearity parameters, respectively. The mechanical contribution of the fibers is excluded under compression.

In this study, the carotid wall tissue was assumed to be nearly incompressible with a Poisson’s ratio value ν = 0.49 (Bäumler et al. 2020). The material parameters c = 0.03574 MPa, $k_{1}$ = 0.0139 MPa, and $k_{2}$ = 13.2, reported by Hariton et al. (2007b) and obtained by fitting the experimental extension and inflation tests data on human carotid wall tissue by Delfino et al. (1997), were used in Eq. (9) and Eq. (10).

To provide an indication of the stiffness of the arterial tissue, the elastic modulus E was calculated from the isotropic component $\overline{\Psi}_{{{\mathrm{iso}}}}$ of the SEF, as follows:

11 $$E = 2 c \left( {1 + \nu } \right)$$

resulting in a value equal to 0.107 MPa. Using E, the bulk modulus ${\mathrm{K}}$ in Eq. (8) was calculated according to the definition:

12 $${\mathrm{K}} = \frac{E}{{3\left( {1 - 2\nu } \right)}}$$

resulting in a value equal to 1.775 MPa. Material properties were assumed to be constant in the carotid wall.

### Prestress of the wall structural domain

The carotid lumen geometries reconstructed from CE-MRA images were assumed to be subjected to end-diastolic luminal pressure, counterbalanced by the arterial-wall internal stresses, i.e., the so-called prestress, as it corresponds to the initial loading state of the arterial wall.

**Fig. 2:**
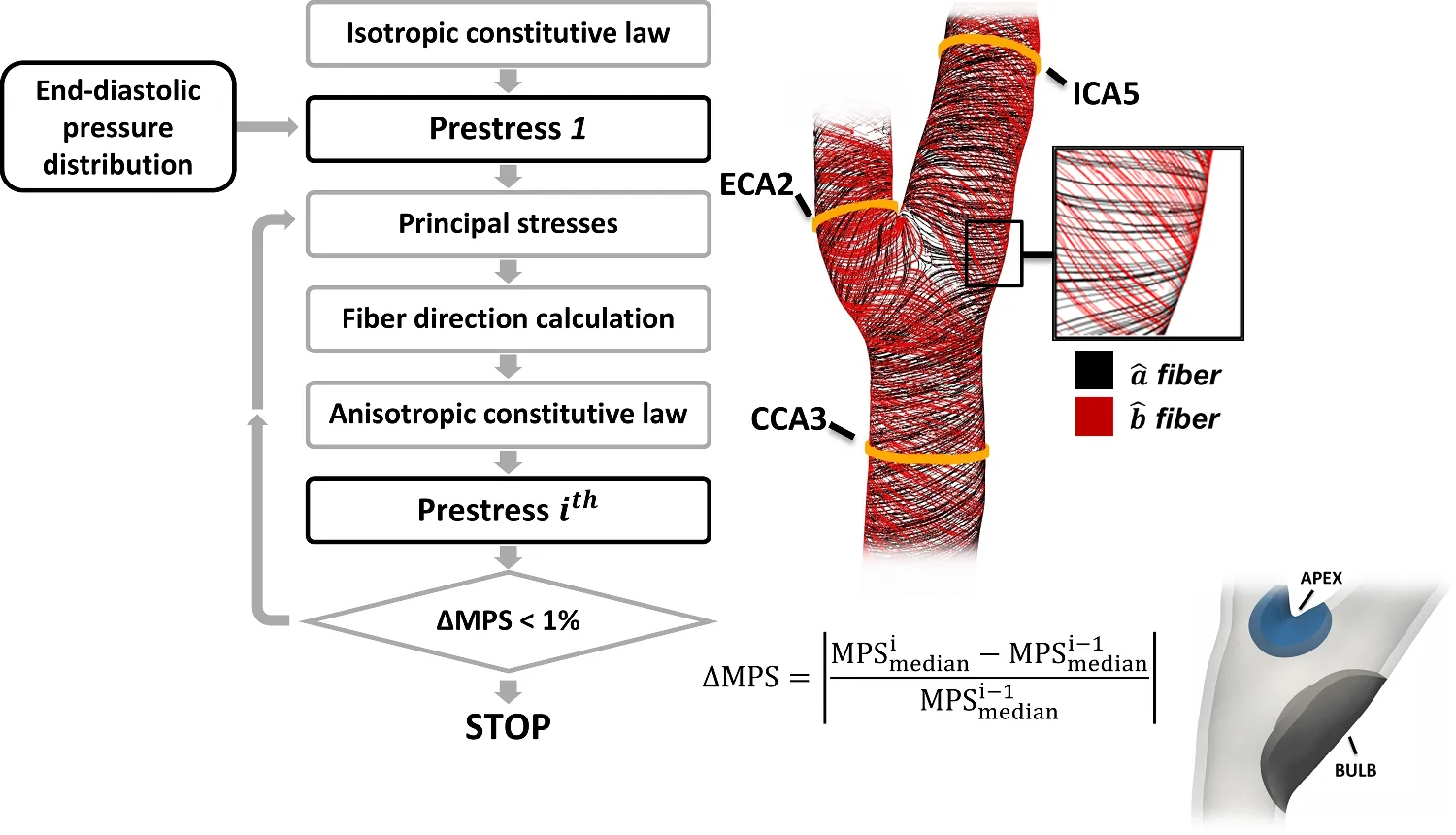
Iterative procedure to determine the initial loading state and the collagen fiber directions. Initially, an isotropic constitutive law was used to obtain the prestress. Then, the direction of the two collagen fiber families was determined based on the maximum principal stress (MPS). An example of fiber directions is shown in the upper right part of the figure, together with the cross-sections located at 3, 5 and 2 radii along the CCA, ICA and ECA, respectively, denoted CCA3, ICA5 and ECA2. These sections delimit the region used for the subsequent quantitative CFD-FSI comparison. The iterative procedure continued until percentage differences in MPS at the apex and carotid bulb (as shown in the bottom right part of the figure) between two consecutive iterations were less than 1%

Briefly, to obtain the prestress, the svFSI solver from the SimVascular package (Zhu et al. 2022) implements a modification of the formulation of the solid problem by employing an additive decomposition of the second Piola–Kirchhoff stress tensor adding an a priori specified prestress tensor (Hsu and Bazilevs 2011). Additionally, the initial loading state was used here to determine the orientation of the two collagen fibers families within the vessel wall. The prestress tensor and the collagen fibers directions were determined according to the procedure represented schematically in Fig. 2 and described in detail below. First, the diastolic luminal pressure acting on the carotid wall was derived from a rigid-wall CFD simulation, where the steady inflow rate at end diastole and outlet resistance tuned to obtain the patient-specific measured flow split between ICA and ECA were imposed as boundary conditions (Bäumler et al. 2020). Next, an initial prestress tensor was obtained by iteratively solving the momentum balance between the CFD-derived diastolic pressure and the internal stresses in the carotid wall while setting the wall displacement to zero, until the carotid wall under the prescribed pressure and subject to prestress reached an equilibrium configuration, as detailed elsewhere (Hsu and Bazilevs 2011; Bäumler et al. 2020). To this end, the following isotropic SEF, which neglects the contribution of the collagen fiber families, was adopted as constitutive equation:

13 $$\overline{\Psi}_{*} = \frac{c}{2} \left( {\overline{{I_{1} }} - 3} \right) + \frac{{k_{1} }}{{k_{2} }} [{\mathrm{e}}^{{k_{2} \left[ {\frac{1}{3}\overline{{I_{1} }} - 1} \right]^{2} }} - 1]$$

In the second step, the first two principal (tensile) Cauchy stresses and their corresponding directions were computed and used to determine the initial orientation of the collagen fiber families. The directions of the two collagen fiber families, defined by the unit vectors ${\hat{\boldsymbol{a}}}$ and ${\hat{\boldsymbol{b}}}$, were defined in terms of their components along the unit vectors $\widehat{{{\boldsymbol{e}}_{1} }}$ and $\widehat{{{\boldsymbol{e}}_{2} }}$, which correspond to the first and second principal directions, respectively. In detail, vectors ${\hat{\boldsymbol{a}}}$ and ${\hat{\boldsymbol{b}}}$ were assumed to form an angle γ (the so-called alignment angle) with respect to the first principal direction $\widehat{{{\boldsymbol{e}}_{1} }}$, so that $\cos \left( {2\gamma } \right) = \hat{\boldsymbol{a}} \cdot \hat{\boldsymbol{b}}$. According to previous studies (Hariton et al. 2007a, b), the alignment angle γ was defined based on the ratio between the magnitudes of the two largest principal stresses $\sigma_{1}$ and $\sigma_{2}$ (Hariton et al. 2007b):

14 $$tg\left( \gamma \right) = \frac{{\sigma_{2} }}{{\sigma_{1} }}$$

15 $$\hat{\user2{a}} = \cos \gamma \widehat{{{\boldsymbol{e}}_{1} }} + \sin \gamma \widehat{{{\boldsymbol{e}}_{2} }}$$

16 $$\hat{\user2{b}} = \cos \gamma \widehat{{{\boldsymbol{e}}_{1} }} - sin\gamma \widehat{{{\boldsymbol{e}}_{2} }}$$

In the subsequent step, the previously computed collagen fiber directions were then incorporated in the anisotropic SEF formulation described by Eq. (10) to calculate a new prestress tensor. From this updated prestress state, collagen fiber directions were recalculated. This iterative process continued until the median values of MPS of the prestress tensor over two regions of the solid domain (the apex and bulb, Fig. 2) differed by less than 1% between two subsequent iterations (Hariton et al. 2007b).

### Comparison of FSI vs. CFD simulations

For the purpose of comparison, CFD simulations assuming rigid walls were carried out using the same inflow and outflow boundary conditions, numerical settings and computational grid employed in the FSI simulations. For each case, the rigid-wall CFD simulation was performed on the end-diastolic carotid bifurcation geometry obtained from the paired FSI simulation. The impact of wall distensibility was evaluated in terms of (i) WSS-based quantities at the luminal surface, and (ii) intravascular hemodynamics. Following previous work (Lee et al. 2008), a quantitative analysis was conducted on the bifurcation region defined by cross-sections located at 3, 5 and 2 radii along the CCA, ICA and ECA, respectively, denoted CCA3, ICA5 and ECA2 (Fig. 2).

Regarding WSS, the luminal distributions of canonical WSS-based quantities were computed, namely time-averaged WSS, TAWSS, and oscillatory shear index, OSI (Table 2). Additionally, WSS topological skeleton features were quantified based on their emerging association with markers of vascular disease at the carotid bifurcation (Morbiducci et al. 2020). In detail, WSS manifolds were identified based on the divergence of the normalized WSS vector field (${{DIV}}_{W}$) (Table 2): negative ${{DIV}}_{W}$ values indicated attracting (stable) manifolds, while positive values indicated repelling (unstable) ones (Mazzi et al. 2020). The variability in the contraction/expansion action exerted by the WSS on the endothelium along the cardiac cycle was quantified in terms of the topological shear variation index, TSVI (Morbiducci et al. 2020; Mazzi et al. 2022) (Table 2). According to previous studies (Lee et al. 2008; Gallo et al. 2018; Morbiducci et al. 2020), threshold values for identifying regions on the luminal surface exposed to disturbed shear were objectively calculated by pooling WSS-based quantities from the 10 rigid models. In detail, the threshold values corresponded to the 20^th^ percentile for TAWSS, and 80^th^ percentile for OSI and TSVI. The resulting threshold values were 0.43 Pa for TAWSS, 0.16 for OSI and 491 m^−1^ for TSVI. Luminal surface area (SA) below (TAWSS) or above (OSI, TSVI) these thresholds were calculated. Then, to factor out the influence of vessel size, the percentage surface areas (%SAs) of the bifurcation regions exposed to disturbed shear were calculated by scaling the surface area extension to the total area of the region bounded by CCA3, ICA5, and ECA2. These %SAs were named TAWSS20, OSI80, and TSVI80, respectively. For the FSI simulations, WSS-based quantities averaged over the cardiac cycle were mapped onto the rigid surface mesh of the corresponding CFD model. Subsequently, pairwise comparisons of SAs derived from CFD and FSI models were quantified in terms of spatial co-localization, using the Similarity Index (SI), defined as:

17 $${\mathrm{SI}} = \frac{{2 \left( {{\mathrm{SA}}_{{j, {\mathrm{CFD}}}} \cap {\mathrm{SA}}_{{j, {\mathrm{FSI}}}} } \right)}}{{{\mathrm{SA}}_{{j, {\mathrm{CFD}}}} \cup {\mathrm{SA}}_{{j,{\mathrm{FSI}}}} }}$$

with *j* = TAWSS20, OSI80, TSVI80. The SI quantifies the spatial co-occurrence of the considered indicators of disturbed shear. It ranges from 0 to 1, where 0 indicates no spatial overlap between the SAs in the two paired simulations, and 1 denotes perfect spatial overlap.

**Table 2:** Definition of hemodynamic descriptors used for the comparison between CFD and FSI simulations

*WSS-based quantities*	
Time-averaged WSS (TAWSS)	${\mathrm{TAWSS}} = \frac{1}{T}\mathop \smallint \limits_{T}^{ } \left| {{\boldsymbol{\tau}}} \right|{{d}}t$
Oscillatory shear index (OSI)	${\mathrm{OSI}} = 0.5 \cdot \left[ {1 - \left( {\frac{{\left| {\mathop \smallint \nolimits_{T}^{ } {\boldsymbol{\tau}} {{d}}t} \right| }}{{\mathop \smallint \nolimits_{T}^{ } \left| {\boldsymbol{\tau}} \right| {{d}}t}}} \right)} \right]$
Divergence of the normalized WSS (*DIV*_*W*_)	${{DIV}}_{W} = \nabla \cdot {\boldsymbol{\tau}_{u}} = \nabla \cdot \left( {\frac{{\boldsymbol{\tau}} }{\left| {\boldsymbol{\tau}} \right|}} \right)$
Topological shear variation index (TSVI)	${\mathrm{TSVI}} = \left[ {\frac{1}{T}\mathop \smallint \limits_{T}^{ } ({{DIV}}_{W} - \overline{{{{DIV}}_{W} }} )^{2} {{d}}t} \right]^{\frac{1}{2}}$
*Intravascular quantities*	
Local normalized helicity (LNH)	${\mathrm{LNH}} = \frac{{\boldsymbol{v}} \cdot {\boldsymbol{\omega}} }{{\left| {\boldsymbol{v}} \right| \cdot \left| {\boldsymbol{\omega}} \right|}}$
Average helicity intensity (h_2_)	${\text{ h}_{2}} = \frac{1}{TV}\mathop \smallint \limits_{T}^{ } \mathop \smallint \limits_{V}^{ } \left| {{\boldsymbol{v}} \cdot {\boldsymbol{\omega}} } \right| {{d}}V {{d}}t$
Average unsigned helical rotation balance (h_4_)	${\mathrm{h}_{4}} = \frac{{\left| {\mathop \smallint \limits_{T}^{ } \mathop \smallint \limits_{V}^{ } {\boldsymbol{v}} \cdot {\boldsymbol{\omega}} dV dt} \right|}}{{\mathop \smallint \limits_{T}^{ } \mathop \smallint \limits_{V}^{ } \left| {{\boldsymbol{v}} \cdot {\boldsymbol{\omega}} } \right| dV dt}}$
Percentage volume of recirculation (%VolRec)	$\% {\mathrm{Vol}}{\mathrm{Rec}}^{i} = 100 \cdot \frac{1}{{V^{i} }} \mathop \sum \limits_{k = 1}^{N} \delta_{k}^{i} V_{k}^{i}$ with $$\delta_{k}^{i} = \left\{ {\begin{array}{*{20}c} {1 \ if \ {v}_{ax,k}^{i} < 0} \\ {0 \ if \ {v}_{ax,k}^{i} \ge 0} \\ \end{array} } \right.$$ and $V^{i} = \mathop \sum \limits_{k =1}^{N} V_{k}^{i}$

${{\boldsymbol{\tau}}}$ is the WSS vector; T is the duration of the cardiac cycle; ${{DIV}}_{W}$ is the divergence of the WSS unit vector ${\boldsymbol{\tau}_{u}}$, and $\overline{{{{DIV}}_{W} }}$ denotes its cycle-averaged value; ${\boldsymbol{v}}$ is the velocity vector; ${\boldsymbol{\omega}}$ is the vorticity vector; V is the volume of the bifurcation delimited by sections CCA3, ICA5, and ECA2; i indicates the *i-th* time point along the cardiac cycle; $V_{k}^{i}$ is the *k-th* elemental volume within the CCA3-ICA5-ECA2 instantaneous volume $V^{i}$; ${{v}}_{ax,k}^{i}$ is the axial velocity component in $V_{k}^{i}$ at time point i; N is the total number of finite volume elements in $V^{i}$

The analysis also included evaluation of the impact of wall distensibility on intravascular hemodynamics. Given the established physiological significance of helical blood flow in the carotid bifurcation and the emerged evidence of its atheroprotective role (Gallo et al. 2012, 2015, 2018; Morbiducci et al. 2016), the intravascular hemodynamics was characterized primarily in terms of helicity content. Firstly, the local normalized helicity, LNH, (Gallo et al. 2012; Morbiducci et al. 2007), defined as the normalized internal product between local velocity and vorticity vectors, was employed to visualize helical fluid structures. As a signed quantity, the visualization of LNH isosurfaces was adopted to distinguish between left-handed and right-handed helical flow patterns within the carotid bifurcation (Gallo et al. 2012). In addition, helical flow was quantitatively evaluated. First, the quantity h_2_ was computed, defined as the time- and volume-averaged norm of the internal product of velocity and vorticity vectors, providing a global measure of helical flow intensity (Gallo et al. 2012, 2018). Second, the unsigned helical rotation balance, denoted as h_4_ (Gallo et al. 2012), was calculated to quantify the relative strength of counter-rotating helical fluid structures (Table 2).

The amount of recirculating flow within the carotid bifurcation was also estimated by applying the scheme previously proposed elsewhere (Martorell et al. 2014; Gallo et al. 2018). Technically, the velocity vector field was projected along the local vessel centerline, computed from the end-diastolic geometry. For each *i-th* time point along the cardiac cycle the finite-element volumes $V_{k}^{i}$ within the CCA3-ICA5-ECA2 volume $V^{i}$ exhibiting a negative axial velocity component ${{v}}_{ax,k}^{i}$ were summed up and then normalized by $V^{i}$ (Table 2). In FSI models, recirculation was evaluated using the relative velocity (${\boldsymbol{v}} - \hat{\user2{v}}$), thereby excluding the mesh velocity component. This computation was performed at each timestep with respect to the instantaneous CCA3-ICA5-ECA2 volume $V^{i}$. The resulting instantaneous ${{\% VolRec}}^{i}$ values were then averaged over the cardiac cycle to obtain the average recirculation volume value ($\% {\mathrm{Vol}}{\mathrm{Rec}}_{{{\mathrm{avg}}}}$).

## Results

### Consistency of CFD and FSI simulations

The physiological relevance of the FSI simulations was assessed by evaluating the maximum cross-sectional area variation at CCA3. Across the analyzed cases, this variation ranged from 9.1% to 20.8%, as detailed in Supplementary Table 4.

An initial comparison between CFD and FSI models was conducted by analyzing the differences in cycle-average ICA/CCA percentage flow ratios, obtained by imposing identical Windkessel boundary conditions in both modeling approaches. The absolute differences between the in vivo MRI-measured and simulated ICA/CCA flow ratios, expressed in terms of median [interquartile range (IQR)] values, were 1.90% [IQR: 1.02% – 2.97%] for CFD, and 3.64% [IQR: 1.00% – 5.19%] for FSI simulations (Supplementary Table 5). The absolute difference between CFD- and FSI-simulated percentage flow ratios was 1.45% [IQR: 0.95% – 3.14%] (Supplementary Table 5).

### CFD vs. FSI simulations: WSS-based quantities

**Fig. 3:**
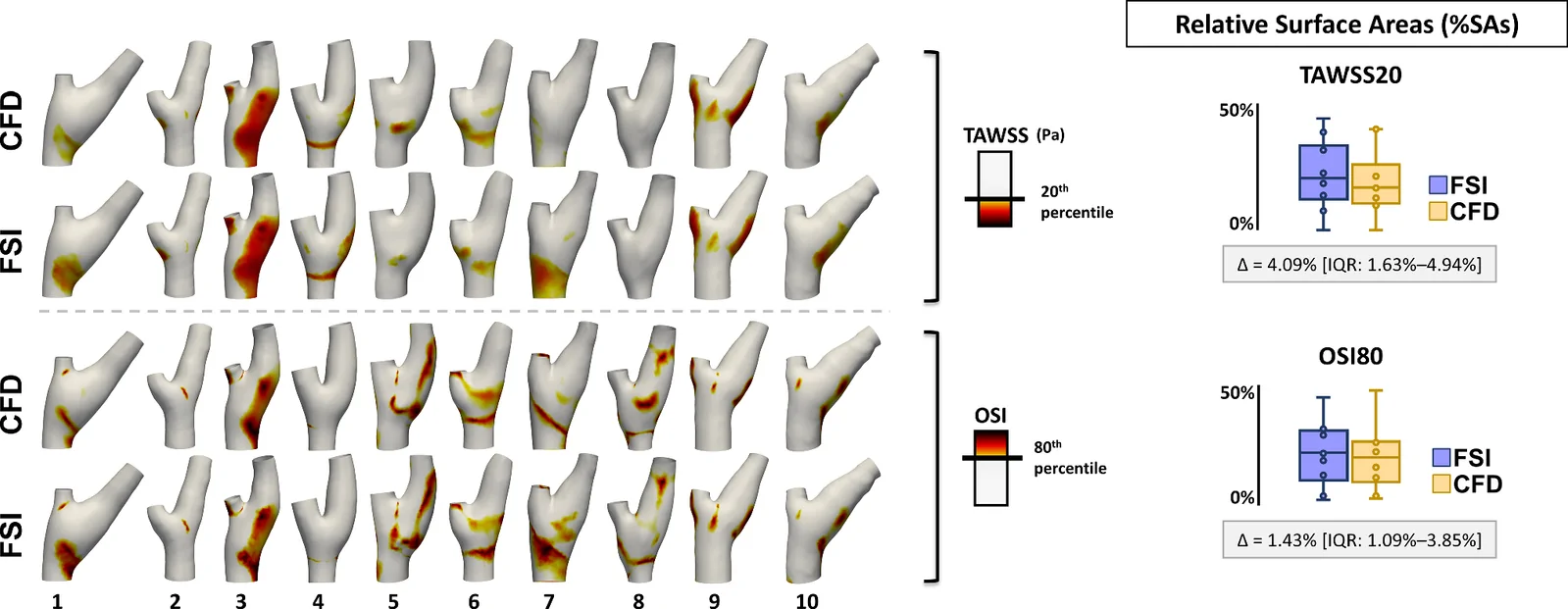
TAWSS and OSI contours highlighting the SAs exposed to low TAWSS (TAWSS20) and high OSI (OSI80) on the diastolic CCA3-ICA5-ECA2 luminal surfaces (left panel). The bar plots of the %SAs values for the CFD-simulated and the FSI-simulated carotid bifurcation models are also displayed (right panel), with the respective absolute differences (Δ)

The SAs exposed to low TAWSS (TAWSS20) and high OSI (OSI80), as simulated by CFD and FSI models, are shown in Fig. 3 for each carotid bifurcation case. Paired CFD- and FSI-simulated absolute differences in %SAs, expressed in terms of median [IQR] values, were 4.09% [IQR: 1.63%–4.94%] for TAWSS20, and 1.43% [IQR: 1.09%–3.85%] for OSI80 (Fig. 3). Case-specific differences are summarized in Supplementary Table 6. The spatial co-localization of disturbed shear indicators between CFD and FSI simulations models, suggested visually by Fig. 3, was confirmed quantitatively by the median SI values, equal to 0.83 [IQR: 0.75–0.87] and 0.79 [IQR: 0.60–0.86], respectively for TAWSS20 and OSI80, as detailed in Supplementary Table 7.

**Fig. 4:**
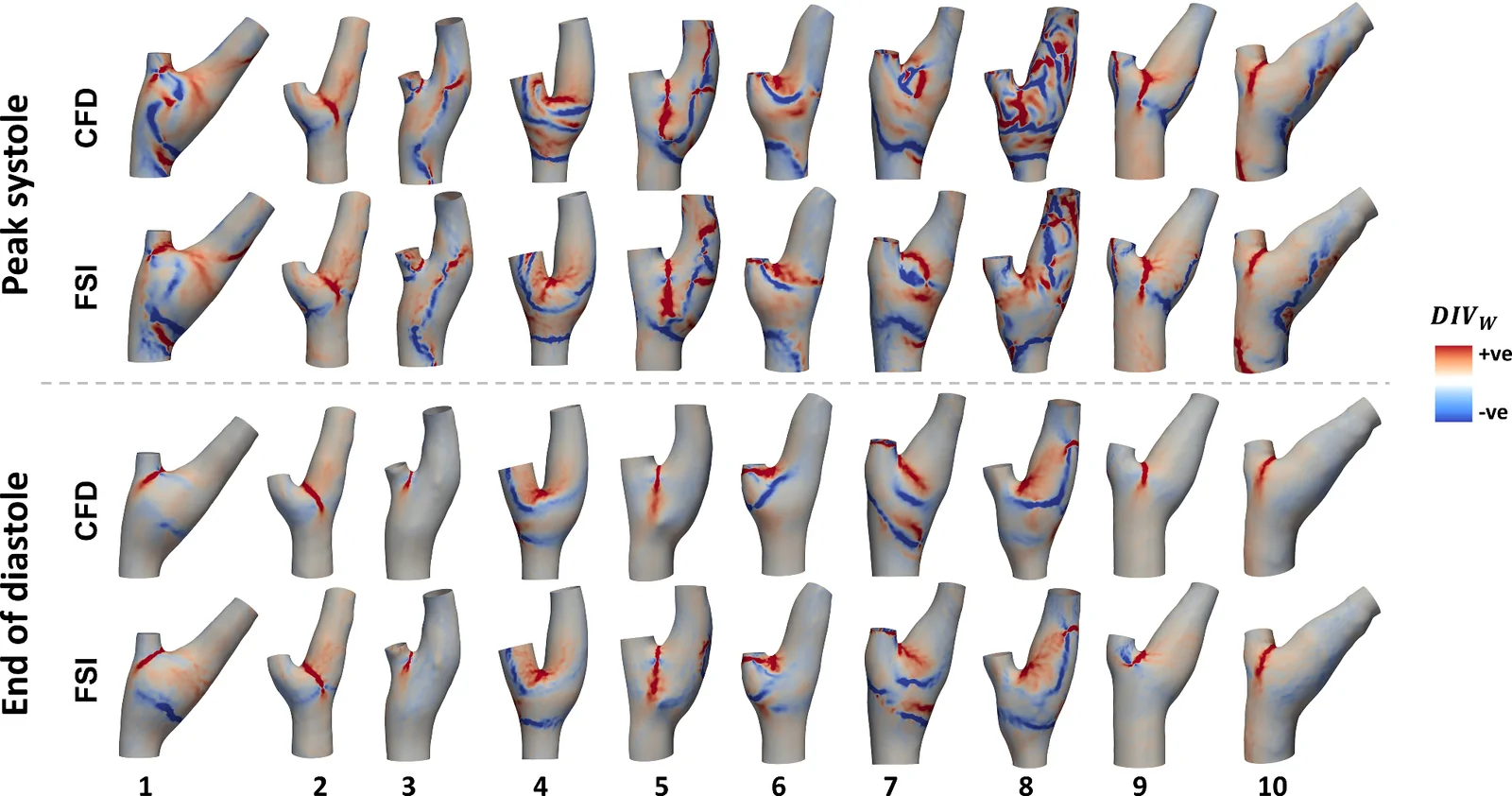
WSS manifolds as identified on the luminal surface of CFD- and FSI-simulated carotid bifurcation models by *DIV*_*W*_ at peak systole (upper panel) and at late diastole (lower panel). Negative values of *DIV*_*W*_ (blue color) identify stable (attracting) manifolds. Positive values of *DIV*_*W*_ (red color) identify unstable (repelling) manifolds

The impact of wall distensibility on near-wall hemodynamics was further examined through analysis of the topological skeleton of the WSS field. The instantaneous stable and unstable manifolds structure, identified through ${{DIV}}_{W}$, was evaluated at two key timepoints along the cardiac cycle, i.e., peak systole and end of diastole. As illustrated in Fig. 4, CFD and FSI models presented markedly similar topological patterns on the luminal surfaces of CFD- and FSI-simulated models. This suggests that carotid bifurcation distensibility introduces minor alterations to the WSS manifolds patterns.

**Fig. 5:**
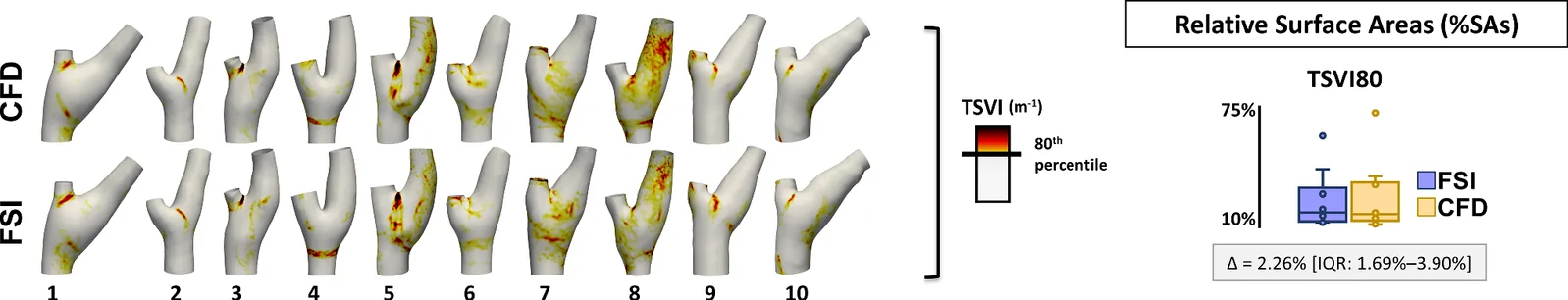
TSVI contours highlighting the SAs exposed to high TSVI (TSVI80) on the diastolic CCA3-ICA5-ECA2 luminal surfaces (left panel). The bar plots of the %SAs values for the CFD-simulated and the FSI-simulated carotid bifurcation models are also displayed (right panel), with the respective absolute differences (Δ)

The effect of wall distensibility on WSS topological skeleton dynamic variability was assessed using TSVI. SAs exposed to high TSVI values (TSVI80) in both CFD- and FSI-simulated models are shown in Fig. 5. Paired absolute differences in %SAs exposed to high TSVI were 2.26% [IQR: 1.69%–3.90%]. Case-specific differences are reported in Supplementary Table 6. The spatial co-localization of TSVI80 regions between CFD and FSI simulations quantified by SI was 0.68 [IQR: 0.67–0.75] (Supplementary Table 7), indicating a substantial co-localization.

### CFD vs. FSI simulations: intravascular flow

**Fig. 6:**
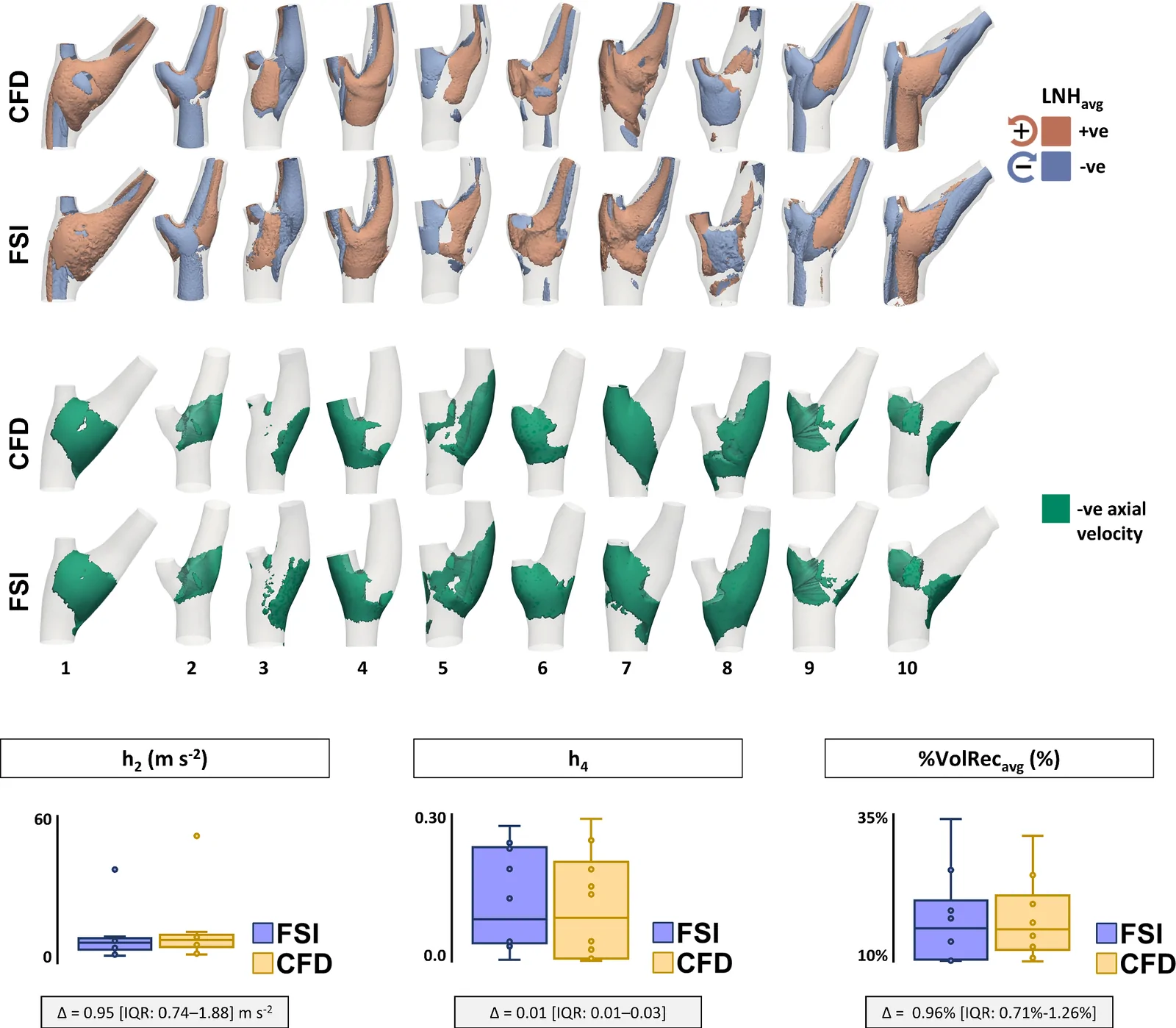
Intravascular hemodynamics features plotted in the diastolic CCA3-ICA5-ECA2 carotid bifurcation volumes. Upper panel: helical right-handed (LNH_avg_ =  + 0.4, red color) and left-handed (LNH_avg_ = -0.4, blue color) structures are reported. Counter-rotating helical flow patterns characterized the flow field of all cases. Mid panel: the volume of recirculating flow, i.e. the volume having negative cycle-averaged axial velocity, is highlighted in green in the diastolic CCA3-ICA5-ECA2 carotid bifurcation volumes. Lower panel: bar plots of h_2_, h_4_, and %VolRec_avg_ values for the CFD-simulated and FSI-simulated carotid bifurcation models, with the respective absolute differences (Δ)

The cycle-averaged LNH visualizations shown in Fig. 6 confirm that, irrespective of wall distensibility, intravascular hemodynamics in ostensibly healthy carotid bifurcations is characterized by the presence of two counter-rotating helical fluid structures, in accordance with previous findings (Gallo et al. 2012, 2015).

Wall distensibility exerted a moderate influence on helicity intensity within the bifurcation region. Specifically, $\mathrm{h}_{2}$ values in FSI simulations models were generally lower compared to those in CFD simulations, with absolute differences of 0.95 [IQR: 0.74–1.88] m·s^−2^ (Fig. 6 and Supplementary Table 8). Wall distensibility had negligible effects on the balance between the counter-rotating helical fluid structures, as highlighted by the comparable $\mathrm{h}_{4}$ values in CFD and FSI simulations, with absolute differences of 0.01 [IQR: 0.01–0.03]). Similarly, the influence of wall distensibility on flow reversal in the CCA3-ICA5-ECA2 region, quantified in terms of ${{\% VolRec}} _{{{\mathrm{avg}}}}$, was limited, with absolute difference between CFD and FSI simulations of 0.96% [IQR: 0.71%–1.26%] (Fig. 6).

## Discussion

In this study, we performed fully coupled two-way FSI simulations incorporating a prestressed fiber-reinforced hyperelastic material and external tissue support to examine the impact of modeling wall distensibility on the hemodynamics of the healthy carotid bifurcation. A paired comparison between FSI and rigid-wall CFD simulations was conducted across 10 ostensibly normal carotid bifurcation models. The analysis focused on (i) WSS-based quantities, which have been linked to early-stage atherosclerosis (Ku et al. 1985; Gallo et al. 2018; Morbiducci et al. 2020), (ii) helicity-based quantities, given the established physiological significance and atheroprotective role of helical blood flow (Gallo et al. 2012, 2018), and (iii) flow reversal, previously linked to an increased likelihood of atherosclerosis initiation and progression (Zarins 1983; Martorell et al. 2014).

While previous studies comparing FSI and CFD simulations of the carotid bifurcation have primarily focused on canonical WSS-based descriptors on a limited number of cases (Bantwal et al. 2021; Aryan et al. 2025; Lopes et al. 2020), the present work is, to our knowledge, the largest to date and the first to extend this comparison to include WSS topological skeleton and intravascular flow features characterizing helical flow and flow reversal. Furthermore, unlike previous FSI studies that neglected the physiological diastolic pre-tensional state of the vessel wall, our approach incorporates the calculation of the prestress tensor. Although the effect of prestressing the arterial wall was not explicitly quantified in the present study, a previous investigation using a fiber-reinforced constitutive law in finite-element structural simulations of an ostensibly healthy human carotid bifurcation demonstrated that although the prestress magnitude was small, it still affected both the stress distribution and wall displacement (Alastrué et al. 2010). Thus, these findings support the inclusion of prestress in our FSI modeling pipeline.

Furthermore, most prior FSI studies on the carotid bifurcation modeled the arterial wall as an isotropic hyperelastic material (Lopes et al. 2020), with the exception of Kamenskiy et al. (2013), who employed a fiber-reinforced formulation to investigate a different context from the present study—specifically, arteriotomy repair strategies following carotid endarterectomy using an idealized average carotid geometry. Importantly, several previous studies have shown that the healthy carotid wall exhibits anisotropic behavior due to the preferred orientation of its structural components—particularly collagen, which is a primary contributor to the anisotropic mechanical response (Hariton et al. 2007a; Alastrué et al. 2010; Kamenskiy et al. 2013). The current approach incorporates the explicit response associated to collagen fibers, enabling a more realistic representation of carotid biomechanics. Maps of wall displacement at peak pressure during the cardiac cycle, confirming the circumferential and axial anisotropic behavior of the carotid wall, are provided in Supplementary Fig. 2. To determine the collagen fiber orientations, a stress-driven methodology, which leverages the iterative procedure for the prestress calculation, was employed here. This approach is based on the hypothesis that collagen fibers align with respect to the local largest Cauchy principal stresses in the initial loading state of the carotid wall. This is consistent with previous studies proposing that collagen fiber directions are oriented to optimize the load bearing capacity of the tissue (Hariton et al. 2007a, b; Alastrué et al. 2010).

The reliability of the FSI simulations was supported by the realistic range of maximum cross-sectional area and diameter variations observed at the CCA3 cross-sections. Overall, the results are broadly consistent with previous FSI studies (Perktold and Rappitsch 1995; Zhao et al. 2000; Lopes et al. 2019; Pozzi et al. 2021) as well as with patient-specific in vivo measurements. Specifically, Crowe et al. (2005) reported MRI-measured maximum area variations at the CCA of 15.1% ± 5.1%, closely align with our result of 13.2% ± 3.4% (Supplementary Table 4). Additionally, ultrasound-based measurements of diameter variation at the CCA by Segers et al. (2004) compare favorably with our results (6.6% ± 2.2% *vs.* 6.4 ± 1.5% of the current study, Supplementary Table 4).

The main findings of this study suggest that the admittedly cumbersome implementation of wall distensibility in FSI simulations generally led to small-to-moderate differences in WSS and intravascular flow patterns compared to rigid-wall CFD. Regarding WSS-based quantities, the extension of SAs exposed to low TAWSS (TAWSS20) as well as high OSI (OSI80) and TSVI (TSVI80) differed less than 4.1% between paired CFD and FSI simulations (specifically, median absolute differences of 4.09% for TAWSS, 1.43% for OSI, and 2.26% for TSVI, Figs. 3 and 4). Notably, these median differences were primarily driven by individual cases (case 7 for TAWSS, case 9 for OSI, and case 8 for TSVI, Supplementary Table 6). In terms of spatial co-localization, high TSVI appeared more sensitive to wall distensibility than low TAWSS or high OSI. Nonetheless, the SI values for TSVI remained high in absolute terms (median SI = 0.68, Supplementary Table 7), indicating substantial overlap between CFD and FSI results. The differences observed between CFD and FSI simulations are in line with previous observations in the ascending aorta, where comparable TSVI patterns were reported when comparing moving- and rigid-wall models (Calò et al. 2023). Conversely, in highly deformable vascular structures such as pulmonary autografts, where large displacements occur, the rigid-wall assumption led to marked differences in TSVI, compared to FSI simulations (Balasubramanya et al. 2024). In the case of the carotid bifurcation, the extent of wall displacements observed here (Supplementary Fig. 2) appears insufficient to significantly alter the distribution of TSVI on the luminal surface. This is further supported by the similar instantaneous distributions of ${{DIV}}_{W}$ seen in paired CFD and FSI simulations at peak systole, when wall displacement is expected to be elevated (with the exception of case 8, and, to a lesser extent, case 7, Fig. 4).

The comparison between CFD and FSI simulations highlighted that wall distensibility also had a moderate impact on intravascular flow characteristics, as quantified by (i) helicity-based quantities, and (ii) flow reversal (Fig. 6). Among the analyzed cases, case 8 consistently exhibited the largest discrepancies between CFD and FSI simulations across all evaluated intravascular flow descriptors (i.e., $\mathrm{h}_{2}$, $\mathrm{h}_{4}$ and ${{\% VolRec}}_{{{\mathrm{avg}}}}$, Fig. 6 and Supplementary Table 8), as well as for the high TSVI region (TSVI80, Fig. 5 and Supplementary Table 6). Moreover, for both CFD and FSI simulations case 8 was also characterized by (i) the highest value of TSVI80 among all cases, and (ii) a null value of TAWSS20, indicating that all TAWSS values in this case were above the threshold used to define the low TAWSS region (Fig. 3 and Supplementary Table 6). Several factors could contribute to these observations. Besides exhibiting a relatively high distensibility at the CCA3 compared to the other cases (Supplementary Table 4), case 8 presents the largest flare at the bifurcation—a known marker of disturbed shear and flow separation, as quantified in previous studies (Gallo et al. 2018). This expansion extends from the region proximal to the bifurcation into the ICA. Additionally, case 8 features the highest cycle-average inlet Reynolds number and the highest heart rate among the considered cases (Supplementary Table 5). The former implies a dynamic WSS field characterized by higher magnitude values, while the latter leads to a shortening of the diastolic phase, i.e. influencing the velocity-dependent cycle-average intravascular quantities.

## Limitations

This study faces possible limitations. Specifically, the three-element Windkessel parameters were tuned using rigid-wall simulations and the resulting parameters were adopted in both CFD and FSI simulations. This choice resulted in differences in the flow repartition between ICA and ECA in paired CFD and FSI simulations (Supplementary Table 5) which could potentially confound the observed differences. For example, case 9 exhibited the largest difference in ICA/CCA flow ratio between CFD and FSI simulations (5.2% of the CCA flow rate), which may partially explain the high difference observed in OSI seen in this case. However, most cases exhibited only minimal variations in the ICA/CCA flow ratio. Windkessel parameters were not recalibrated for the 3D FSI simulations because this would require a computationally expensive iterative procedure based on 3D FSI modeling, as the adopted 1D/0D framework cannot represent the full 3D HGO wall mechanics or the external tissue support.

Uncertainty in modeling the mechanical behavior of the arterial wall can arise from two main sources: the selection of parameters in the wall material model and the wall thickness. In this study, the material parameters were not subject-specific but were selected based on histological data reported in literature (Hariton et al. 2007b). Material properties and wall thickness were assumed constant throughout the entire geometry for each subject. The assumption of constant material properties is consistent with previous studies on healthy carotid bifurcations (Hariton et al. 2007b; Lopes et al. 2020). However, to increase model personalization, wall thickness was set by using subject-specific wall thickness measurements taken at the ICA bulb. Convergence of the prestress–fibers orientation was iteratively assessed using the relative change in the median MPS within two biomechanically relevant regions (i.e., bifurcation apex and bulb) as a pragmatic and sufficiently accurate criterion (Supplementary Fig. 3), despite representing a weak convergence measure. Moreover, the adopted prestress approach provides only the initial (diastolic) stress state, without an associated deformation (Mourato et al. 2024). The impact of this limitation has been previously investigated in FSI simulations of ascending thoracic aorta aneurysm geometries (Mourato et al. 2024) that compared the prestress approach adopted here with an inverse approach that estimates the zero-pressure geometry (Raghavan et al. 2006). When comparing the two approaches, comparable hemodynamic results were obtained in terms of blood pressure and TAWSS. Differences were primarily observed in OSI and RRT, with varying magnitudes across the investigated cases. Given that wall deformations in the present carotid bifurcation cases are smaller than in the thoracic aorta cases considered in that study (Mourato et al. 2024), we expect the impact of the adopted prestress approach on hemodynamic results to be minimal.

While the use of CE-MRA acquisitions for carotid geometry reconstruction offers advantages such as the availability from large carotid disease studies and the ease and reliability of the geometry segmentation, it also presents some limitations. Specifically, in regions of low velocities and/or small lumen size, dilution of the contrast agent during acquisition may result in vessel edge blurring, causing lumen area underestimation (Khan et al. 2013). Such area underestimation may in turn lead to velocity overestimations in our simulations, since the imposed inlet blood flow rates were separately obtained from in vivo PC-MRI measurements. Moreover, the CE-MRA derived carotid lumen geometries were assumed here to correspond to an end-diastolic configuration. This assumption may affect the estimation of the end-diastolic luminal pressure in the prestress calculation procedure (Fig. 2), but its impact on the CFD *vs*. FSI comparison is expected to be limited.

## Conclusions

Rigid-wall CFD simulations adequately capture the biologically and clinically relevant hemodynamic characteristics of the carotid bifurcation, including both established and emerging WSS-based and intravascular quantities. This suggests that rigid-wall CFD approaches may prove adequate for practical clinical applications, where computational costs and turnaround time of simulations are of paramount importance. However, despite inherent higher computational costs and added uncertainties, FSI models offer invaluable advantages such as the computation of structural quantities enabling to explore the synergistic relationship between these quantities and hemodynamic stresses on the endothelium (Tziotziou et al. 2025), thereby potentially providing insights into the effect of these biomechanical stimuli on carotid atherosclerosis initiation.

## Supplementary Information

Below is the link to the electronic supplementary material.Supplementary file1 (DOCX 5431 KB)

## Data Availability

No datasets were generated or analysed during the current study.
