# Supplementary material for: Influence of wall distensibility on local hemodynamics at the normal carotid bifurcation: a fluid–structure interaction study
Source: Biomech Model Mechanobiol. 2026 Jul 21;25(4):85. doi: 10.1007/s10237-026-02103-4 (PMC13388534; doi:10.1007/s10237-026-02103-4)
Supplement: Supplementary file 1 — Supplementary file1 (DOCX 5431 KB) [file 10237_2026_2103_MOESM1_ESM.docx]

**Influence of wall distensibility on local hemodynamics at the normal carotid bifurcation: a fluid-structure interaction study**

**Supplementary material**

Sara Zambon ^a^, Valentina Mazzi ^a^, Karol Calò ^a^, Mariachiara Arminio ^a^, Sabrina Nocerino ^a^,

Claudio Chiastra ^a^, David A. Steinman ^b^, Umberto Morbiducci ^a^, Diego Gallo ᶧ^a^

^a^ Polito^BIO^Med Lab, Department of Mechanical and Aerospace Engineering, Politecnico di Torino, Turin, Italy

^b^ Biomedical Simulation Laboratory, Department of Mechanical & Industrial Engineering, University of Toronto, Toronto, ON Canada

#
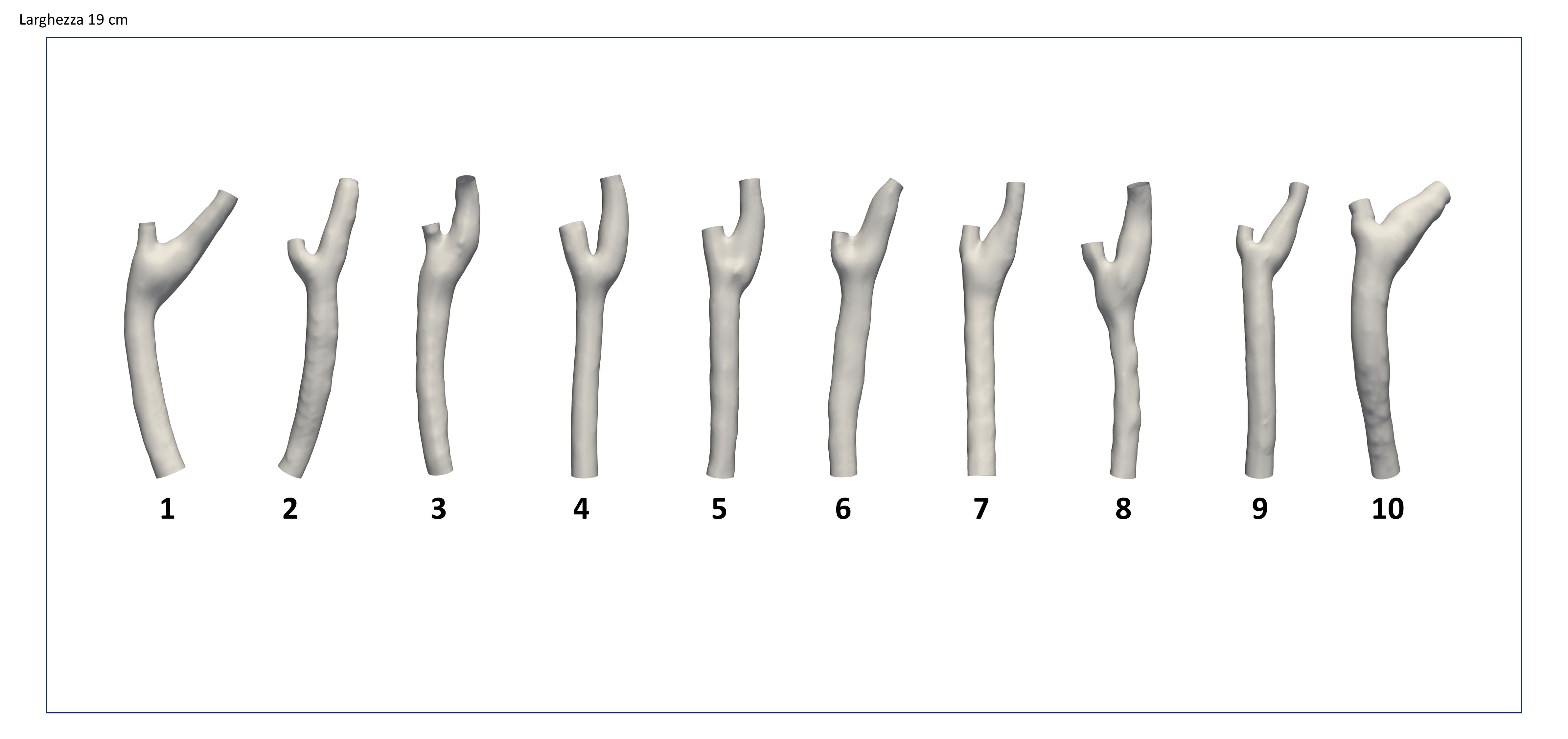
Supplementary Figure 1 - Lumen geometries used for CFD and FSI simulations. Geometries were clipped at least fifteen, seven and three radii from the bifurcation.


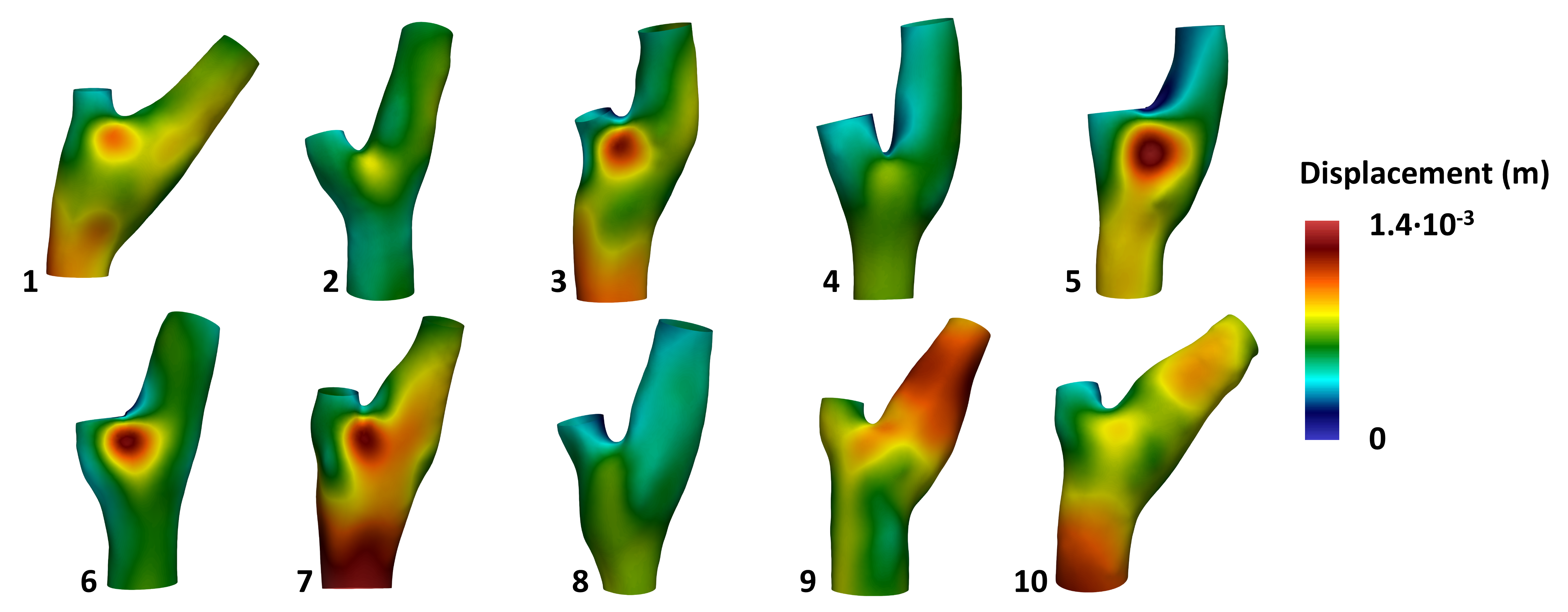


Supplementary Figure 2 – Surface maps of wall displacement at the time of peak pressure during the cardiac cycle for the ten cases in the CCA3-ICA5-ECA2 region.


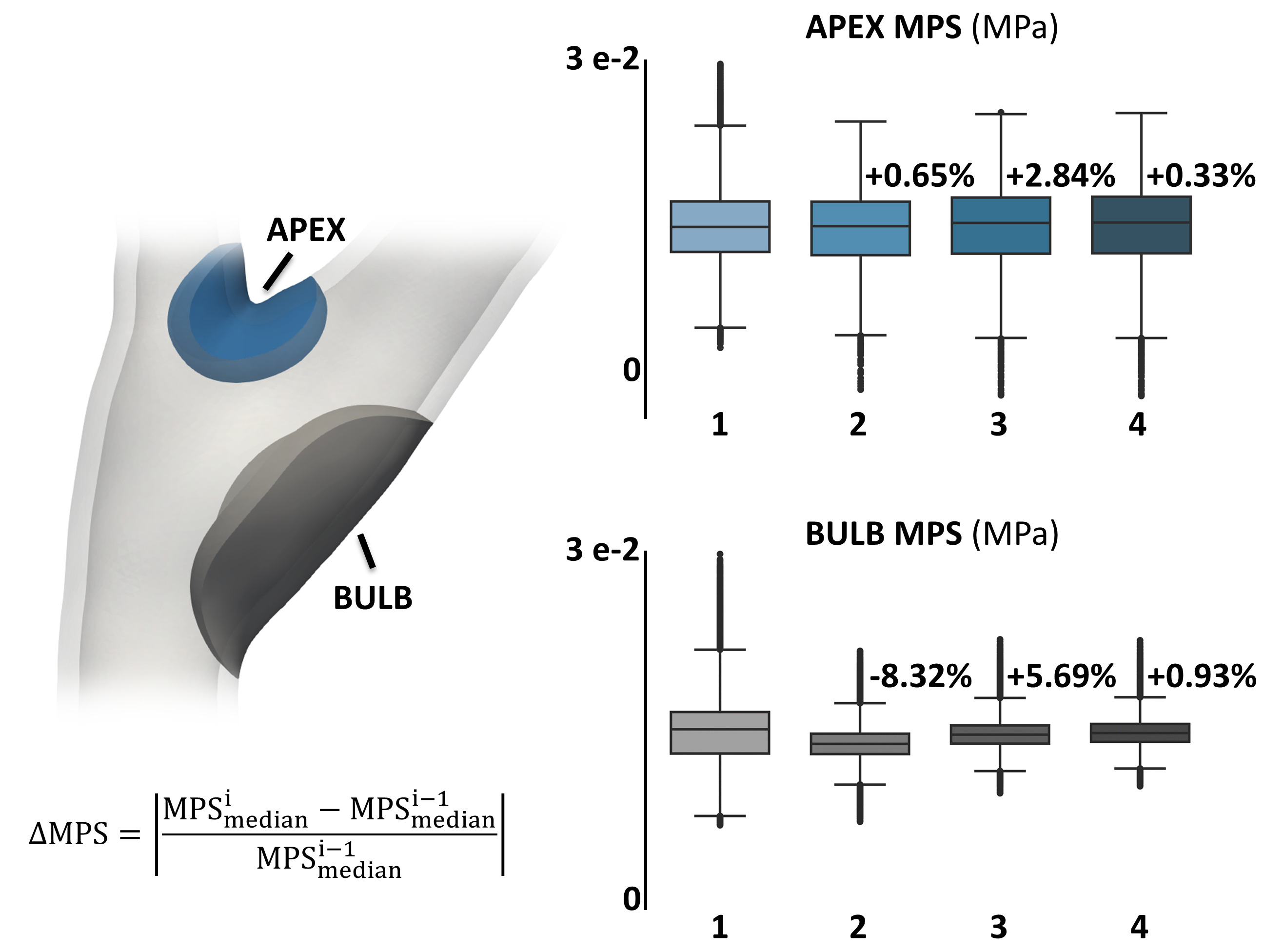


**Supplementary Figure 3** – Prestress iterations in a representative model (model #1) achieved convergence (i.e., ΔMPS<1%) in four iterations. Boxplots of MPS values in the apex and bulb regions highlight that the median is representative of the MPS distribution. The median is represented by an horizontal line; the box extends from lower to upper quartile, while the whiskers indicate 5^th^ and 95^th^ percentile values.

Supplementary Table 1 - Fluid domain grid independence analysis across five refinement levels. Reported quantities include the maximum element size, total number of elements, surface area–averaged topological shear variation index (TSVI), and cycle- and volume-averaged local normalized helicity (LNH). The surface-averaged TSVI was considered over the bifurcation region delimited by cross-sections CCA3–ICA5–ECA2 while the cycle- and volume-averaged LNH was considered over the CCA3–ICA5–ECA2 volumetric region.

|  | **Maximum element size** (10^-3^ m) | **Total number of elements** (∙10^6^) | **TSVI** (10^2^ m^-1^) | **Cycle- and volume-average LNH** (-) |
| --- | --- | --- | --- | --- |
| **Finest** | 0.12 | 8.52 | 1.49 | 0.042 |
| **Finer** | 0.14 | 6.50 | 1.68 | 0.040 |
| **Intermediate** | 0.17 | 3.20 | 2.23 | 0.040 |
| **Coarser** | 0.22 | 1.54 | 2.11 | 0.037 |
| **Coarsest** | 0.27 | 0.77 | 2.15 | 0.038 |

Supplementary Table 2 - Solid domain grid independence analysis across four refinement levels. Reported quantities include maximum element size, total number of elements, and spatially averaged maximum principal stress (MPS) and maximum principal strain (MPStrain). Averages are computed over the solid domain excluding the flow extension regions (“Whole domain”), as well as over the bifurcation apex and bulb sub-regions (“Apex Region” and “Bulb Region”).

|  | **Maximum element size** (10^-3^ m) | **Total number of elements** (∙10^6^) | **Average MPS** (10^4^ Pa) | | | **Average MPStrain** | | |
| --- | --- | --- | --- | --- | --- | --- | --- | --- |
|  |  |  | **Whole domain** | **Apex** | **Bulb** | **Whole domain** | **Apex** | **Bulb** |
| **Finest** | 0.17 | 3.50 | 2.26 | 3.01 | 3.24 | 0.02 | 0.03 | 0.03 |
| **Finer** | 0.28 | 1.00 | 2.22 | 3.01 | 3.22 | 0.02 | 0.03 | 0.03 |
| **Coarser** | 0.39 | 0.52 | 2.20 | 2.91 | 3.24 | 0.02 | 0.03 | 0.03 |
| **Coarsest** | 0.70 | 0.24 | 2.13 | 2.60 | 3.12 | 0.02 | 0.02 | 0.02 |

Supplementary Table 3 - Table containing the selected three-elements Windkessel model parameters for all the ICA and ECA outlets for each of the 10 carotid models considered. The proximal resistance is indicated with Rp, the distal one with Rd. Capacitance is indicated with C. Resistances are expressed in Pa·s·m^-3^ and capacitances in m^3^·Pa^-1^.

| **Case number** | **ECA** | | | **ICA** | | |
| --- | --- | --- | --- | --- | --- | --- |
|  | **Rp**  (Pa·s·m^-3^) | **C**  (m^3^ ·Pa^-1^) | **Rd**  (Pa·s·m^-3^) | **Rp**  (Pa·s·m^-3^) | **C**  (m^3^ ·Pa^-1^) | **Rd**  (Pa·s·m^-3^) |
| **1** | 6.09 · 10^8^ | 5.39 · 10^-11^ | 4.05 · 10^9^ | 3.66 · 10^8^ | 1.26·10^-10^ | 2.43 · 10^9^ |
| **2** | 4.61 · 10^8^ | 4.92 · 10^-11^ | 3.46 · 10^9^ | 3.43 · 10^8^ | 1.15·10^-10^ | 2.58 · 10^9^ |
| **3** | 1.33 · 10^9^ | 5.36 · 10^-11^ | 8.21 · 10^9^ | 4.75 · 10^8^ | 1.25·10^-10^ | 2.94 · 10^9^ |
| **4** | 1.76 · 10^9^ | 4.97 · 10^-11^ | 5.44 · 10^9^ | 5.91 · 10^8^ | 1.16·10^-10^ | 1.83 · 10^9^ |
| **5** | 1.66 · 10^8^ | 2.11 · 10^-10^ | 2.89 · 10^9^ | 1.92 · 10^8^ | 9.03·10^-11^ | 3.36 · 10^9^ |
| **6** | 6.82 · 10^8^ | 4.63 · 10^-11^ | 9.83 · 10^9^ | 1.57 · 10^8^ | 1.08·10^-10^ | 2.27 · 10^9^ |
| **7** | 1.33 · 10^8^ | 5.05 · 10^-11^ | 1.04 · 10^10^ | 2.26 · 10^8^ | 1.18·10^-10^ | 1.77 · 10^9^ |
| **8** | 1.11 · 10^8^ | 1.43 · 10^-10^ | 3.46 · 10^9^ | 1.17 · 10^8^ | 6.12·10^-11^ | 3.63 · 10^9^ |
| **9** | 4.77 · 10^8^ | 7.18 · 10^-11^ | 9.54 · 10^9^ | 1.25 · 10^8^ | 1.68·10^-10^ | 2.51 · 10^9^ |
| **10** | 9.28 · 10^8^ | 4.85 · 10^-11^ | 5.39 · 10^9^ | 3.97 · 10^8^ | 1.13·10^-10^ | 2.31 · 10^9^ |

Supplementary Table 4 - Cross-sectional area and diameter variations at CCA3 obtained from FSI simulations of the 10 carotid bifurcations under study. Area (or diameter) variations were calculated as the difference between the systolic and diastolic CCA3 area (or diameter), divided by the diastolic CCA3 area (or diameter).

| **FSI case number** | **Area variation at CCA3 (%)** | **Diameter variation at CCA3 (%)** |
| --- | --- | --- |
| **1** | 20.8 | 9.9 |
| **2** | 11.0 | 5.4 |
| **3** | 13.3 | 6.5 |
| **4** | 11.7 | 5.7 |
| **5** | 9.1 | 4.5 |
| **6** | 15.8 | 7.6 |
| **7** | 14.2 | 6.8 |
| **8** | 14.2 | 6.9 |
| **9** | 10.9 | 5.3 |
| **10** | 10.9 | 5.3 |

Supplementary Table 5 – Reynolds number at the inlet (calculated as the ratio of the product of density, cycle-averaged velocity and inlet diameter over dynamic viscosity), percentage ICA/CCA flow ratio (%) as measured by PC-MRI, and resulting percentage ICA/CCA flow ratio for both CFD and FSI simulations of the 10 carotid bifurcations under study.

| **Case number** | **Inlet Reynolds number** | **Heart rate (bpm)** | **Measured ICA/CCA flow ratio (%)** | **Simulated ICA/CCA flow ratio (%)** | |
| --- | --- | --- | --- | --- | --- |
|  |  |  |  | **CFD** | **FSI** |
| **1** | 472 | 68.6 | 62.6 | 62.3 | 65.9 |
| **2** | 489 | 53.1 | 57.6 | 56.7 | 58.2 |
| **3** | 329 | 59.5 | 72.5 | 74.5 | 72.3 |
| **4** | 513 | 61.7 | 74.2 | 75.0 | 76.4 |
| **5** | 499 | 80.7 | 46.4 | 51.0 | 52.4 |
| **6** | 414 | 74.7 | 80.4 | 83.5 | 84.3 |
| **7** | 492 | 65.1 | 85.2 | 87.0 | 85.6 |
| **8** | 565 | 80.7 | 51.1 | 53.7 | 55.4 |
| **9** | 295 | 49.3 | 78.0 | 82.1 | 87.3 |
| **10** | 443 | 77.2 | 69.8 | 71.5 | 75.3 |

Supplementary Table 6 – Relative percentage surface areas (%SAs) values for the considered WSS-based quantities (TAWSS, OSI and TSVI) obtained from CFD and FSI simulations. For each quantity, the absolute difference Δ is also reported.

| **Case number** | **Descriptor** | **%SA** | | **Δ (%)** |
| --- | --- | --- | --- | --- |
|  |  | **CFD** | **FSI** |  |
| 1 | TAWSS | 9.8% | 13.8% | 4.0% |
|  | OSI | 11.7% | 12.7% | 1.1% |
|  | TSVI | 11.2% | 12.6% | 1.4% |
| 2 | TAWSS | 17.1% | 18.6% | 1.5% |
|  | OSI | 3.7% | 3.7% | 0.1% |
|  | TSVI | 7.6% | 9.5% | 1.9% |
| 3 | TAWSS | 40.3% | 44.5% | 4.2% |
|  | OSI | 28.5% | 30.2% | 1.7% |
|  | TSVI | 7.7% | 8.7% | 1.0% |
| 4 | TAWSS | 21.5% | 23.5% | 2.0% |
|  | OSI | 2.5% | 1.9% | 0.6% |
|  | TSVI | 12.9% | 10.4% | 2.5% |
| 5 | TAWSS | 12.8% | 7.7% | 5.2% |
|  | OSI | 49.6% | 46.6% | 3.0% |
|  | TSVI | 35.4% | 39.5% | 4.1% |
| 6 | TAWSS | 18.3% | 14.0% | 4.3% |
|  | OSI | 23.4% | 22.2% | 1.2% |
|  | TSVI | 14.3% | 16.4% | 2.0% |
| 7 | TAWSS | 11.0% | 31.9% | 20.9% |
|  | OSI | 22.9% | 31.8% | 9.0% |
|  | TSVI | 30.4% | 25.0% | 5.4% |
| 8 | TAWSS | 0.0% | 0.0% | 0.0% |
|  | OSI | 27.0% | 22.9% | 4.1% |
|  | TSVI | 72.2% | 58.8% | 13.3% |
| 9 | TAWSS | 40.3% | 39.1% | 1.2% |
|  | OSI | 16.2% | 32.9% | 16.7% |
|  | TSVI | 10.4% | 8.7% | 1.6% |
| 10 | TAWSS | 16.8% | 22.8% | 6.0% |
|  | OSI | 18.0% | 19.1% | 1.2% |
|  | TSVI | 14.6% | 18.0% | 3.4% |

Supplementary Table 7 – Similarity Index (SI) for the three WSS-based quantities quantifying the co-localization of SAs. Case 8 had null SA for TAWSS, as shown in Supplementary Table 4.

| **Case number** | **TAWSS** | **OSI** | **TSVI** |
| --- | --- | --- | --- |
| **1** | 0.82 | 0.87 | 0.75 |
| **2** | 0.86 | 0.76 | 0.79 |
| **3** | 0.95 | 0.90 | 0.67 |
| **4** | 0.92 | 0.48 | 0.69 |
| **5** | 0.56 | 0.90 | 0.75 |
| **6** | 0.75 | 0.82 | 0.62 |
| **7** | 0.48 | 0.58 | 0.67 |
| **8** | --- | 0.53 | 0.78 |
| **9** | 0.87 | 0.64 | 0.64 |
| **10** | 0.83 | 0.82 | 0.67 |

Supplementary Table 8 – Values of the computed intravascular flow features (h_2_, h_4_, and %VolRec_avg_) obtained from CFD and FSI simulations. For each quantity, the absolute difference Δ is also reported.

| **Case number** | **Descriptor** | **CFD** | **FSI** | **Δ** |
| --- | --- | --- | --- | --- |
| 1 | h_2_ (m/s^2^) | 10.9 | 8.6 | 2.3 |
|  | h_4_ (-) | 0.02 | 0.03 | 0.006 |
|  | %VolRec_avg_ (%) | 20.1% | 18.7% | 1.3% |
| 2 | h_2_ (m/s^2^) | 7.4 | 6.5 | 0.9 |
|  | h_4_ (-) | 0.04 | 0.04 | 0.003 |
|  | %VolRec_avg_ (%) | 10.6% | 10.6% | 0.1% |
| 3 | h_2_ (m/s^2^) | 3.1 | 2.7 | 0.4 |
|  | h_4_ (-) | 0.18 | 0.22 | 0.041 |
|  | %VolRec_avg_ (%) | 12.9% | 10.9% | 2.0% |
| 4 | h_2_ (m/s^2^) | 10.2 | 9.1 | 1.0 |
|  | h_4_ (-) | 0.01 | 0.04 | 0.031 |
|  | %VolRec_avg_ (%) | 16.8% | 17.5% | 0.7% |
| 5 | h_2_ (m/s^2^) | 10.2 | 9.5 | 0.7 |
|  | h_4_ (-) | 0.01 | 0.004 | 0.002 |
|  | %VolRec_avg_ (%) | 24.5% | 25.3% | 0.8% |
| 6 | h_2_ (m/s^2^) | 6.9 | 5.6 | 1.3 |
|  | h_4_ (-) | 0.15 | 0.18 | 0.034 |
|  | %VolRec_avg_ (%) | 13.3% | 14.3% | 1.0% |
| 7 | h_2_ (m/s^2^) | 12.5 | 10.4 | 2.1 |
|  | h_4_ (-) | 0.1 | 0.1 | 0.008 |
|  | %VolRec_avg_ (%) | 19.8% | 18.7% | 1.1% |
| 8 | h_2_ (m/s^2^) | 54.6 | 39.9 | 14.7 |
|  | h_4_ (-) | 0.002 | 0.04 | 0.037 |
|  | %VolRec_avg_ (%) | 30.9% | 33.6% | 2.8% |
| 9 | h_2_ (m/s^2^) | 2.7 | 2.2 | 0.5 |
|  | h_4_ (-) | 0.28 | 0.27 | 0.014 |
|  | %VolRec_avg_ (%) | 11.1% | 10.7% | 0.4% |
| 10 | h_2_ (m/s^2^) | 7.9 | 7.0 | 0.9 |
|  | h_4_ (-) | 0.24 | 0.23 | 0.005 |
|  | %VolRec_avg_ (%) | 14.6% | 13.7% | 0.9% |
